# Profiling of RNA Viruses in Biting Midges (*Ceratopogonidae*) and Related Diptera from Kenya Using Metagenomics and Metabarcoding Analysis

**DOI:** 10.1128/mSphere.00551-21

**Published:** 2021-10-13

**Authors:** Solomon K. Langat, Fredrick Eyase, Wallace Bulimo, Joel Lutomiah, Samuel O. Oyola, Mabel Imbuga, Rosemary Sang

**Affiliations:** a Department of Biochemistry, Jomo Kenyatta University of Agriculture and Technology, Nairobi, Kenya; b Centre for Virus Research, Kenya Medical Research Institute, Nairobi, Kenya; c Institute of Biotechnology Research, Jomo Kenyatta University of Agriculture and Technology, Nairobi, Kenya; d Department of Emerging Infectious Diseases, United States Army Medical Research Directorate—Africa, Nairobi, Kenya; e Department of Biochemistry, University of Nairobi, Nairobi, Kenya; f International Livestock Research Institute, Nairobi, Kenya; Icahn School of Medicine at Mount Sinai

**Keywords:** metagenomics, metabarcoding, biting midges, RNA viruses

## Abstract

Vector-borne diseases (VBDs) cause enormous health burden worldwide, as they account for more than 17% of all infectious diseases and over 700,000 deaths each year. A significant number of these VBDs are caused by RNA virus pathogens. Here, we used metagenomics and metabarcoding analysis to characterize RNA viruses and their insect hosts among biting midges from Kenya. We identified a total of 15 phylogenetically distinct insect-specific viruses. These viruses fall into six families, with one virus falling in the recently proposed negevirus taxon. The six virus families include *Partitiviridae*, *Iflaviridae*, *Tombusviridae*, *Solemoviridae*, *Totiviridae*, and *Chuviridae*. In addition, we identified many insect species that were possibly associated with the identified viruses. *Ceratopogonidae* was the most common family of midges identified. Others included *Chironomidae* and *Cecidomyiidae*. Our findings reveal a diverse RNA virome among Kenyan midges that includes previously unknown viruses. Further, metabarcoding analysis based on COI (cytochrome *c* oxidase subunit 1 mitochondrial gene) barcodes reveal a diverse array of midge species among the insects used in the study. Successful application of metagenomics and metabarcoding methods to characterize RNA viruses and their insect hosts in this study highlights a possible simultaneous application of these two methods as cost-effective approaches to virus surveillance and host characterization.

**IMPORTANCE** The majority of the viruses that currently cause diseases in humans and animals are RNA viruses, and more specifically arthropod-transmitted viruses. They cause diseases such as dengue, West Nile infection, bluetongue disease, Schmallenberg disease, and yellow fever, among others. Several sequencing investigations have shown us that a diverse array of RNA viruses among insect vectors remain unknown. Some of these could be ancient lineages that could aid in comprehensive studies on RNA virus evolution. Such studies may provide us with insights into the evolution of the currently pathogenic viruses. Here, we applied metagenomics to field-collected midges and we managed to characterize several RNA viruses, where we recovered complete and nearly complete genomes of these viruses. We also characterized the insect host species that are associated with these viruses. These results add to the currently known diversity of RNA viruses among biting midges as well as their associated insect hosts.

## INTRODUCTION

Vector-borne diseases (VBDs) cause significant health and economic burden all over the world, with the tropical and subtropical regions bearing the heaviest burden. They account for more than 17% of all infectious diseases and are associated with more than 700,000 deaths every year (1, 2). Pathogens that cause VBDs are transmitted by arthropod vectors such as mosquitoes, sandflies, ticks, and biting midges (2, 3). Among these arthropod-transmitted pathogens, viruses account for a disproportionately high number of emerging human pathogens, with RNA viruses alone constituting the highest proportion, approximately 37% of all emerging human pathogens (4). The resurgence and spread of known and re-emerging arthropod-borne RNA viruses around the globe are now widely reported, with devastating consequences; yellow fever, dengue, chikungunya, Zika fever, Rift Valley fever, and Crimean-Congo hemorrhagic fever are the most common (5, 6). Biting midges are vectors of a range of human and livestock pathogens (7, 8). Three of the diseases transmitted by these vectors are currently listed by World Organization for Animal Health (OIE) as notifiable diseases, namely, bluetongue, African horse sickness, and epizootic hemorrhagic disease (9). Midges are generally small insects (approximately 1 to 3 mm in length) belonging to different families within the suborder Nematocera in the order Diptera (10). These families include *Ceratopogonidae* (biting midges), *Chironomidae* (nonbiting midges), and *Cecidomyiidae* (gall midges). *Ceratopogonidae* is the most important family because it contains members that are medically important vectors of disease-causing pathogens like *Mansonella* sp. parasites and viruses such as bluetongue, Oropouche, African horse sickness, epizootic hemorrhagic disease, and Schmallenberg viruses (8, 10, 11).

The response and mitigation strategies for fighting vector-borne diseases are currently dependent on surveillance programs. These programs allow early detection and control of vector-borne diseases as well as identification of any invasive vector species (12, 13). For insect vectors, routine surveillance approaches often involve identification of trapped insect vectors using morphological traits and subsequent screening for pathogens using cell culture and molecular detection methods (14). The challenge with these traditional approaches is that they are time-consuming and difficult to implement, especially when large numbers of specimens are to be processed. The advent and further development of high-throughput sequencing (HTS) technologies have provided powerful tools with enormous potential to overcome these challenges. In this study, we used HTS-based sequencing methods, metagenomics and metabarcoding. Metagenomics is an unbiased approach to sequencing of all the DNA or cDNA in a given sample (15, 16). This method has proven quite useful and has led to rapid progress in virus discovery, including identification of novel pathogens that have been implicated in major outbreaks (17). Metabarcoding, on the other hand, is a method of performing massively parallel sequencing of mixed biological samples that combines the HTS technologies with the traditional DNA barcoding method (18). This strategy enables the generation of a large number of individual barcode sequences for the various insect specimens pooled in a single reaction. Analysis of the barcode sequences generated allows species identification of insects in the given pool (18). The cytochrome *c* oxidase subunit 1 mitochondrial gene (COI) has become the marker of choice for most DNA barcoding studies. The COI marker is often preferred because of its ability to sufficiently discriminate between closely related species, particularly vertebrate and invertebrate species (19–21). Additionally, this target is widely represented in reference databases, with millions of COI reference sequences currently available in public databases (22). Adoption of third-generation sequencing technology allows further improvement of metabarcoding method, with long-read sequencing providing the advantage of higher resolution as a result of the sequencing of longer DNA fragments. In this study, we applied metagenomics and metabarcoding techniques to analyze field-collected specimens of midges obtained from different sites in Kenya. We sought to detect and characterize RNA viruses harbored by these midges and identify their associated vector species based on their COI markers.

## RESULTS

### RNA viruses identified in biting midges.

**FIG 1:**
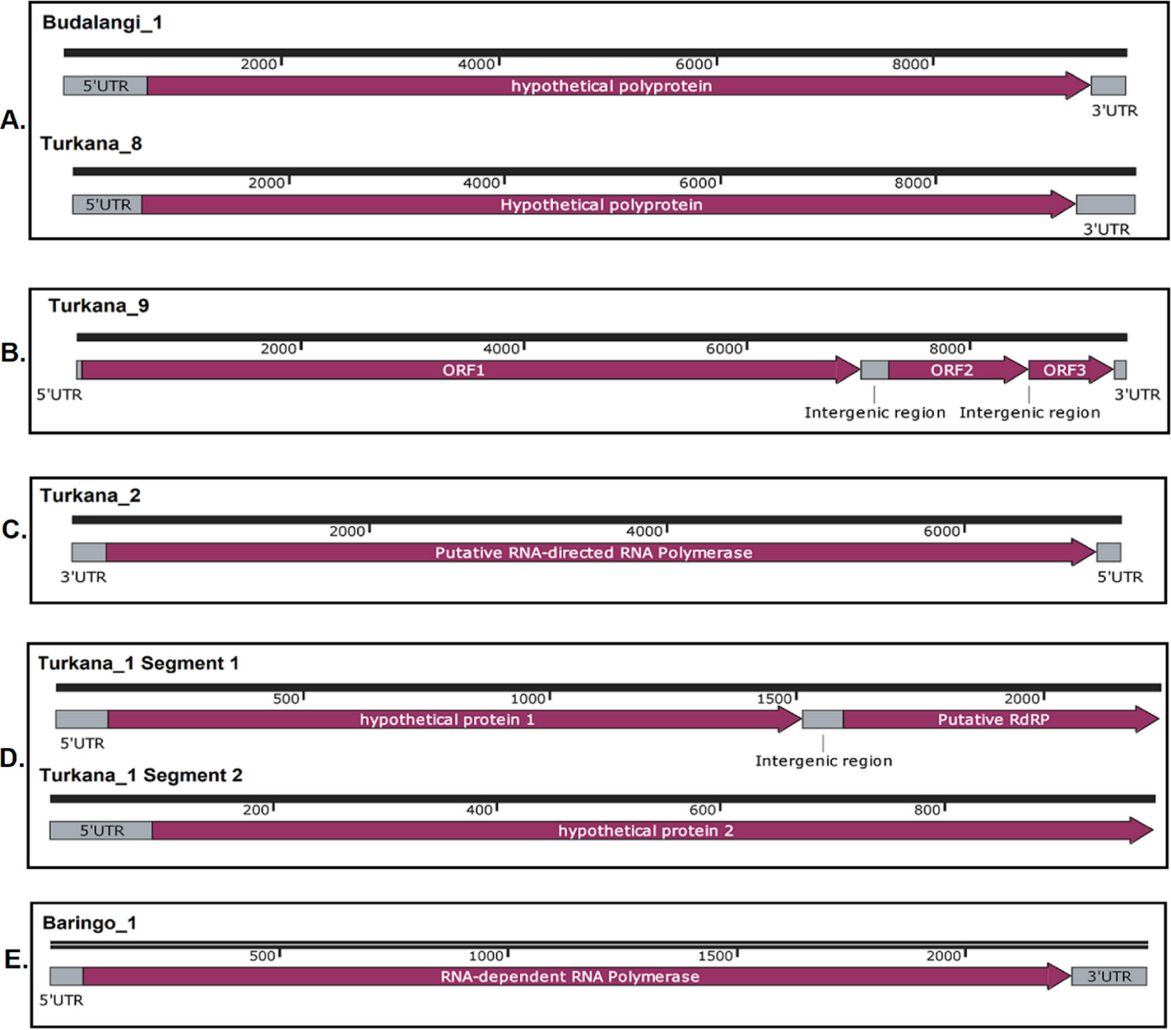
Genome architectures of the representative genomes of complete iflavi-like viruses (A), complete novel negevirus genome (B), partial *Chuviridae* genome showing the RNA-dependent RNA polymerase coding segment (C), a novel *Solemoviridae* virus with the two putative segments (D), and the representative partial *Partitiviridae* genome showing the RNA-dependent RNA polymerase coding segment (E). Similar organization was observed in the other *Partitiviridae* genomes obtained in the study.

A total of 3,351 midge specimens were processed in this study. Of these, 1,063 originated from Turkana and 892 were from Baringo, while Isiolo, Budalangi, and Kacheliba yielded 640, 600, and 156 specimens, respectively. These specimens were used to create bulk pools representing each of the five sites, which were then subjected to high-throughput sequencing. Sequence assembly and analysis led to generation of 15 distinct virus genomes, identified based on the presence of the RNA-dependent RNA polymerase (RdRp) gene, which is the hallmark of all RNA viruses (23, 24). The RdRp gene of the identified viruses was found to be 38.81% to 97.7% similar to previously sequenced viruses available in GenBank. Among the 15 viruses identified, 10 were positive-sense RNA (+ssRNA) viruses, with viruses in the Picornavirales order forming the majority. Viruses in the order *Picornavirales* included 5 that were similar to those in the family *Iflaviridae* and 1 which was similar to those in the family *Picornaviridae*. Turkana_5, which was obtained from a pool of midges from Turkana, was 85.62% similar to Heliconius erato iflavirus (Table 1), an iflavivirus detected in *Heliconius* butterflies in Costa Rica (25). On the other hand, Isiolo_1, which was obtained from a pool of midges from Isiolo, showed a 97.7% similarity to Culex iflavi-like virus 4 which was previously obtained from *Culex* sp. mosquitoes in the United States (26). Two viruses from Budalangi and Turkana, specifically, Budalangi_1 and Turkana_8, showed high similarity to the Redbank virus (Table 1), with 70.84% and 66.3% similarity, respectively, to this previously identified virus. Redbank virus is an unclassified iflavi-like virus recently identified in mosquito fecal microbiota in Australia (27). Kacheliba_1 was the other iflavi-like virus that had relatively low similarity to published sequences. It had 46.6% similarity to Hubei picorna-like virus 38 (NC_033201.1). Also falling in the order *Picornavirales* was Turkana_10, which was identified from a pool of midges from Turkana. This virus had 84.27% similarity to the Boghill Burn virus, an unclassified virus in the family *Picornaviridae* which was identified in *Bombus* sp. bees in Scotland (28). All 6 viruses in the order *Picornavirales* had genome architectures similar to those of other viruses in this order. They have a single polyprotein flanked by 3′ and 5′ untranslated regions (UTRs), and the genome length of these viruses is approximately 9 kb (Fig. 1).

**TABLE 1:** Viruses identified in this study

Strain	Site	Family classification	Accession no.	Closest hit	% identity (RdRp; aa)
Turkana_1	Turkana	*Solemoviridae*	MF893251.1	Medway virus	59.05
Turkana_2	Turkana	*Chuviridae*	KX924630.1	Chuvirus Mos8Chu0	42.39
Turkana_3	Turkana	Tombus-like	KX235518.1	Diaphorina citri associated C virus	55.24
Turkana_4	Turkana	*Totiviridae*	MK440653.1	Lindangsbacken virus	50.79
Turkana_5	Turkana	*Iflaviridae*	NC_024016.1	Heliconius erato iflavirus	85.62
Turkana_6	Turkana	*Partitiviridae*	LC533398.1	Lichen partiti-like RNA virus	46.14
Turkana_7	Turkana	Partiti-like	KX884215.1	Hubei partiti-like virus 45	52.74
Turkana_8	Turkana	Iflavi-like	MN784069.1	Redbank virus	66.3
Turkana_9	Turkana	Negevirus	MT344121.1	Sandewavirus dungfly1	39.13
Turkana_10	Turkana	Picorna-like	MH614292	Boghill Burn virus	84.27
Baringo_1	Baringo	Partiti-like	MF344586.1	Araticum virus	56.01
Budalangi_1	Budalangi	Iflavi-like	MN784065.1	Redbank virus	70.84
Isiolo_1	Isiolo	*Iflaviridae*	NC_040574.1	Culex Iflavi-like virus 4	97.7
Kacheliba_1	Kacheliba	Iflavi-like	NC_033201.1	Hubei picorna-like virus 38	46.37
Kacheliba_2	Kacheliba	*Partitiviridae*	JX658566.1	Grapevine partitivirus	51.61

Other +ssRNA viruses identified in this study include *Solemoviridae*, *Tombusviridae*, and a Negevirus-like virus. Turkana_1 showed similarity to members of the unclassified family *Solemoviridae*. Specifically, it had 59.05% similarity to the Medway virus. *Solemoviridae* family members have been known to be nonsegmented +ssRNA viruses (29). However, similar to recently described sobemo-like viruses, such as Atrato sobemo-like virus 1 and Hubei sobemo-like virus 48 (24), Turkana_1 contained two segments. Further, we also obtained a 1,142-bp sequence belonging to a virus in the family *Tombusviridae*. Turkana_3 had 55.24% similarity to Diaphorina citri-associated C virus. We also identified a Negevirus-like virus, Turkana_9, from a pool of midges from Turkana. Turkana_9 had considerably low similarity to other negeviruses available in GenBank. More specifically, it had 39.13% similarity to Sandewavirus dungfly 1, a negevirus obtained from dung fly in the Arctic Yellow River Station (30). Despite this low similarity, however, the genome architecture of Turkana_9 was similar to that of other negeviruses, with three open reading frames (ORFs) separated by two intergenic regions of various lengths (Fig. 1).

In this study, we also identified one negative-sense RNA (−ssRNA) virus specifically falling in the recently described family *Chuviridae* (31). Turkana_2 had 42.39% similarity to Chuvirus Mos8Chu0 (Table 1). Chuvirus Mos8Chu0 (KX924630.1) is a bisegmented virus belonging to the family Chuviridae, and it was obtained from Culiseta minnesotae mosquitoes. In this study, however, we obtained only a partial sequence corresponding to the entire L segment of Chuvirus Mos8Chu0. This segment contains a single ORF which is the putative RNA-dependent RNA polymerase gene (Fig. 1).

Other viruses identified in this study are five double-strand RNA (dsRNA) viruses that include *Totiviridae* and *Partitiviridae*. Turkana_4 is a *Totiviridae* member which was identified in a pool of midges from Turkana. The virus showed a 50.79% similarity to the Lindangsbacken virus, which is an unclassified *Totiviridae* member. The four *Partitiviridae* viruses detected in this study originated from Turkana, Kacheliba, and Baringo, and these viruses were highly diverse, with a significantly low similarity of 46.14% to Lichen partiti-like virus for Turkana_6 and a higher similarity of 56.01% to Araticum virus for Baringo_1. The other two viruses included Turkana_7, which had 52.74% similarity to Hubei partiti-like virus 45, and Kacheliba_2, which had 51.61% similarity to Grapevine partitivirus. *Partitiviridae* contains viruses with two segments, dsRNA1 and dsRNA2 (32). In this study, however, we obtained only the segment corresponding to the dsRNA1 segment whose ORF codes for the RNA-dependent RNA polymerase. This finding was similar for all four *Partitiviridae* viruses obtained in the study.

### Phylogenetic analysis of the identified RNA viruses.

**FIG 2:**
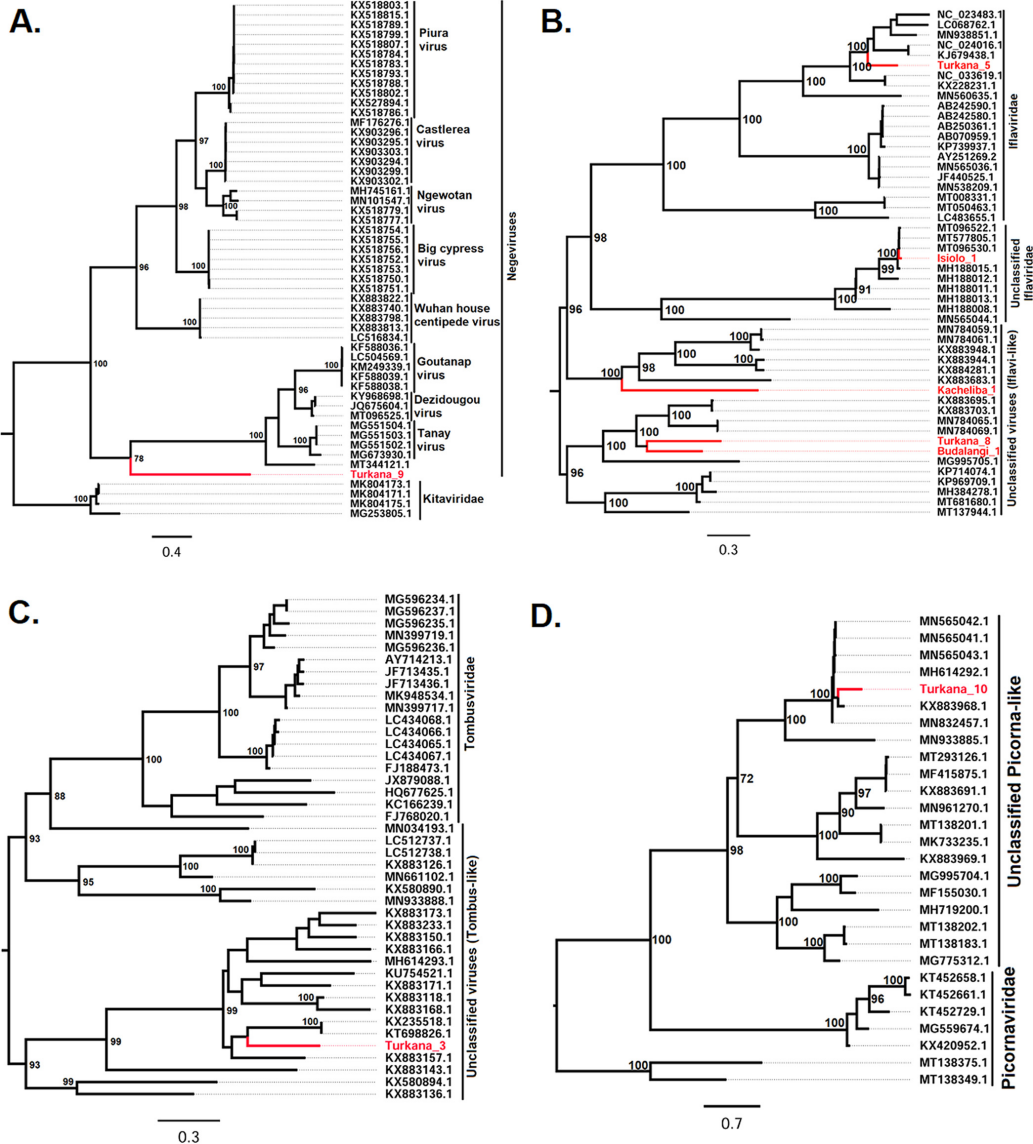
Midpoint rooted phylogenies for negeviruses (A), *Iflaviridae* (B) *Tombusviridae* (C), and *Picornaviridae* (D) and related sequences. The trees were inferred based on 1,000 bootstrap replicates and an approximate-likelihood-ratio test. Confidence values are shown in the tree nodes, and the sequences obtained from the study are in red.

**FIG 3:**
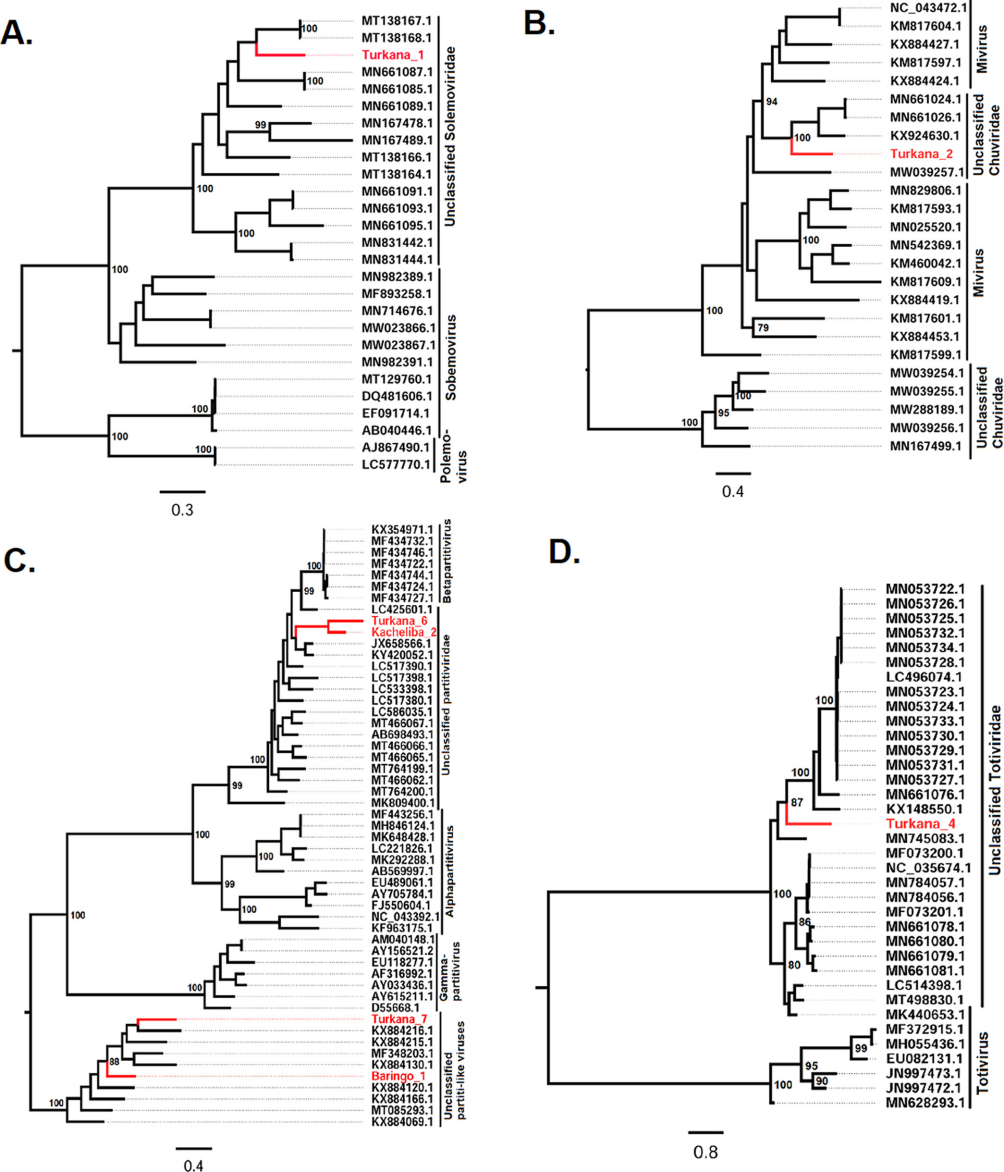
Midpoint rooted phylogenies for *Solemoviridae* (A), *Chuviridae* (B), *Partitiviridae* (C), and *Totiviridae* (D) and related sequences. The trees were inferred based on 1,000 bootstrap replicates and an approximate-likelihood-ratio test. Confidence values are shown in the tree nodes, and the sequences obtained from the study are in red.

Phylogenetic analysis of the newly discovered viruses with other closely related viruses available in GenBank placed 7 of the new viruses in five different families and one in the recently proposed taxon Negevirus (33). More specifically, the six families that these identified viruses fall into or are related to include *Partitiviridae*, *Iflaviridae*, *Tombusviridae*, *Solemoviridae*, *Totiviridae*, and the recently described family *Chuviridae*. Except for Turkana_5, which clustered with iflaviruses (Fig. 2), the majority of the other identified viruses showed high similarity and clustered with diverse virus strains which are yet to be classified within the specific RNA virus families. More specifically, Turkana_6 and Kacheliba_2 clustered with unclassified members of the family *Partitiviridae* (Fig. 3), Isiolo_1 clustered with unclassified members of the family *Iflaviridae* (Fig. 2), Turkana_1 clustered with unclassified members of the family *Solemoviridae*, Turkana_4 clustered with unclassified members of the family *Totiviridae*, and Turkana_2 clustered with unclassified members of the family *Chuviridae* (Fig. 3).

Eight of the identified viruses clustered with diverse virus strains that are yet to be classified into specific families. These included partiti-like, iflavi-like, picorna-like, and tombus-like viruses. Turkana_7 and Baringo_1 clustered with unclassified Partiti-like viruses (Fig. 3); Kacheliba_1, Turkana_8, and Budalangi_1 clustered with unclassified iflavi-like viruses; Turkana_3 clustered with unclassified tombus-like viruses; and Turkana_10 clustered with unclassified picorna-like viruses (Fig. 2). Turkana_9 was closely related and clustered with negeviruses (Fig. 2), forming a single clade with Sandewavirus, Tanay virus, Dezidougou virus, and Goutanap virus, all of which are insect-specific viruses (ISVs).

### Community composition of the midge pools.

Metabarcoding analysis resulted in diverse reads that were assigned to different families of midges, including *Chironomidae*, *Ceratopogonidae*, and *Cecidomyiidae*. All the reads in the Baringo pool were assigned to the family *Ceratopogonidae*, with 65.46, 19.81, and 13.26% of the reads being assigned to Culicoides leucostictus, Culicoides pycnostictus, and Culicoides nivosus, respectively. Similarly, all the reads from the Budalangi site were classified as *Ceratopogonidae* and specifically as *Culicoides leucostictus*. At the Kacheliba site, all the reads identified were classified as *Cecidomyiidae*. All the reads showed similarity to unclassified members within the family *Cecidomyiidae* (Table 2). Isiolo and Turkana sites had relatively diverse reads that were classified as different midge species. The family *Ceratopogonidae* accounted for 44.07%, while *Cecidomyiidae* accounted for 21.03% of the reads at the Isiolo site. The remaining fraction were assigned to the *Chironomidae*. In Turkana, reads assigned to *Ceratopogonidae* were common, with 68.49%. The proportions of reads assigned to *Chironomidae* and *Cecidomyiidae* in Turkana were 25.64 and 5.88%, respectively.

**TABLE 2:** Community composition of the different vector pools for each of the sites

Site	No. of specimens^*a*^	Family	Species	Sequence abundance	Fraction of COI reads (%)	OTU abundance^*b*^
Isiolo	640 (114)	*Cecidomyiidae*	*Cecidomyiidae* sp.	637	21.03	21
		*Ceratopogonidae*	Culicoides leucostictus	260	8.58	2
			Culicoides oxystoma	33	1.09	1
			Culicoides similis	124	4.09	2
			*Forcipomyia* sp.	918	30.31	4
		*Chironomidae*	*Ablabesmyia* sp.	53	1.75	3
			*Chironomidae* sp.	343	11.32	8
			*Polypedilum* sp.	501	16.54	9
			*Tanytarsus* sp.	160	5.28	1

Baringo	892 (873)	*Ceratopogonidae*	Culicoides bedfordi	109	1.46	7
			Culicoides leucostictus	4,871	65.46	17
			Culicoides nivosus	987	13.26	4
			Culicoides pycnostictus	1,474	19.81	8

Turkana	1,063 (884)	*Cecidomyiidae*	*Cecidomyiidae* sp.	212	5.88	8
		*Ceratopogonidae*	Culicoides kingi	260	7.21	6
			*Culicoides leucostictus*	1,010	27.99	9
			*Culicoides* sp.	102	2.83	1
			*Culicoides nivosus*	354	9.81	6
			Culicoides schultzei	745	20.65	14
		*Chironomidae*	*Ablabesmyia* sp.	119	3.3	2
			*Microchironomus* sp.	806	22.34	5

Kacheliba	156 (6)	*Cecidomyiidae*	*Cecidomyiidae* sp.	1110	100	36

Budalangi	600 (600)	*Ceratopogonidae*	*Culicoides leucostictus*	4940	100	13

a The total number of specimens processed for each site is given, with the number of specimens morphologically classified as *Culicoides* sp. in parentheses.

b Number of consensus sequences obtained after sequence clustering.

The specimen sequences obtained were classified into operational taxonomic units (OTUs) (see Materials and Methods). Overall, we obtained a total of 187 OTUs belonging to the different families of midges. The Turkana and Isiolo sites had 51 OTUs each, while Baringo and Kacheliba had 36 OTUs each (Table 2). The Budalangi site had only 13 OTUs, despite a relatively high number of specimens processed as well as of total reads obtained (Table 2). In this study, the findings were generally consistent with the number of specimens that were processed in each of the sites (Table 2). The species richness of the five different sites was variable, with Isiolo and Turkana having relatively diverse species of midges. The insects in Isiolo included *Ceratopogonidae* comprising *Culicoides leucostictus*, Culicoides oxystoma, Culicoides similis, and unclassified *Forcipomyia* sp. The *Chironomidae* family insects obtained in Isiolo included *Ablabesmyia* sp., *Polypedilum* sp., *Tanytarsus* sp., and others that remain unclassified within the family *Chironomidae*. We also identified 21 OTUs in Isiolo that had high similarities to unclassified members of the family *Cecidomyiidae*. In Turkana, we identified members of *Ceratopogonidae* that included Culicoides kingi, *Culicoides leucostictus*, *Culicoides nivosus*, Culicoides schultzei, and unclassified *Culicoides* sp. In addition, *Chironomidae* family OTUs were identified that included *Ablabesmyia* sp. and *Microchironomus* sp. The Baringo and Budalangi sites had OTUs that were all assigned to *Ceratopogonidae*. *Culicoides leucostictus* was identified in both of these sites. In addition, the Baringo site had Culicoides bedfordi, *Culicoides nivosus*, and *Culicoides pycnostictus*. At the Kacheliba site, all the OTUs identified belonged to the family *Cecidomyiidae*. More specifically, these OTUs showed high similarity to unclassified members of the family *Cecidomyiidae*.

## DISCUSSION

High-throughput screening and timely surveillance of viruses and insect vectors are critical for detecting vector-borne diseases. The methods that are currently applied in surveillance and screening of these diseases are dependent on cell culture, serology, and molecular detection methods (34). These methods are quite laborious and they are also limited, given that they can identify only viruses that can be cultured using currently available tools as well as those that have previously been isolated and diagnostic methods developed for them. In this study, we applied metagenomics and metabarcoding approaches to characterize RNA viruses and their associated insect hosts. Even though no known pathogenic viruses were detected in this study, we were able to detect numerous RNA viruses, some of which can be classified as novel. Further, several host species were detected in the pools from which the RNA viruses were identified, providing an important avenue for determining the possible association of detected viruses with their hosts.

The metagenomic approach employed in this study allowed the characterization of several RNA viruses found among midges from five sites in Kenya. These identified viruses add to the currently existing biodiversity of midge-borne viruses. More specifically, sequencing of these field-collected midges allowed us to genetically characterize up to 15 RNA viruses. The majority of these are novel viruses with low similarity thresholds, with as low as 39.13% amino acid similarity to existing viruses. Only Isiolo_1, Turkana_5, and Turkana_10 showed higher similarity to existing viruses, with 97.7%, 85.62%, and 84.27% amino acid similarities, respectively. It should, however, be noted that in this study, we did not carry out the taxonomic classification of the identified viruses to the species level. The International Committee on Taxonomy of Viruses (ICTV) establishes various criteria for the classification of virus species, which often differ depending on virus group, and these criteria include information other than genetic sequences of the virus (35). Further studies should, therefore, be carried out in order to classify the identified viruses to their respective species.

The majority of the viruses identified in this study are ISVs, and none of them is known to be pathogenic to vertebrate hosts. However, even though they are unlikely to be associated with diseases, studies have shown that ISVs can potentially influence the vector competence of arthropod vectors by interfering with their vectorial capacity for pathogenic viruses, possibly due to competitive inhibition (36–38). These viruses, therefore, can act as important biocontrol agents in the transmission of pathogenic viruses. Additionally, identification of these viruses may fill important gaps in the phylogeny of viruses and provide important information to studies aimed at understanding the origin and evolution of pathogenic viruses (39, 40).

Several viruses identified in this study belong to families known to be associated with different hosts other than arthropods. These include *Tombusviridae*, *Solemoviridae*, *Partitiviridae*, *Picornaviridae*, and *Totiviridae*. The families *Tombusviridae* and *Solemoviridae*, for instance, contain viruses that have plants as their natural hosts. The family *Picornaviridae*, on the other hand, contains viruses known to infect only the vertebrates. *Partitiviridae* and *Totiviridae* contain viruses whose natural hosts are known to be quite diverse. Fungi and plants are known natural hosts of *Partitiviridae*, while fungal and protozoan parasites are known to be the natural hosts of viruses in the family *Totiviridae*. Our findings, therefore, suggest that these viruses could also be associated with midges. However, it is also possible that this observation may have been a result of our sample processing strategy. The sample processing in this study involved homogenization of the entire invertebrate specimens. Therefore, some of the viruses detected may have originated from undigested food, gut microflora, or even parasites that may have been present in the invertebrates at the time of processing. This may be true for members of the family *Ceratopogonidae*, whose feeding sources are known to be quite diverse (41–43). It would not, therefore, be surprising if some of the viruses detected have their origin in other hosts other than midges.

Insect community composition identified in the current study is quite diverse. Our metabarcoding approach was able to identify numerous species of midges in each site. The fractions of the reads specific for each of the species identified in this study were quite variable in each of the sites in the study (Table 2). Considering the sensitivity issues associated with HTS as well as primer biases, these observations cannot reliably be used as estimates of the relative abundance of each of the identified species (44, 45). Nonetheless, the identification of a given species in a pool of midges is reason enough for it to be considered a possible host of the viruses identified in the study. This is because the identified virus may have originated from either of the species of midges in the pool, irrespective of their abundance. These findings, therefore, provide us with a unique opportunity to infer the possible hosts of the detected viruses, using methods such as co-occurrence networks (46). Such an approach would, however, require sequencing of multiple pools from a given locality to use in the network and also to help in improving the accuracy of this method. Additionally, approaches to reducing PCR bias can be considered in order to improve the accuracy of host-virus association methods. Some of the approaches to reducing PCR bias include *in silico* testing of primers before use, the use of multiple sets of primers, and the use of PCR-free shotgun sequencing pipelines (44, 47, 48). Future studies should, therefore, consider some of these requirements in order to definitively associate the identified viruses with their insect hosts.

Metagenomics and metabarcoding methods used in this study are potentially cost-effective approaches to arbovirus and insect surveillance. These approaches have the advantage of analyzing hundreds to thousands of insect specimens in a single pool. Traditional methods, on the other hand, would process between 25 and 50 specimens per pool. Further, the classical DNA barcoding method for species identification and confirmation often processes a single specimen at any given time. Thus, the costs and labor are dramatically reduced when metagenomics coupled with metabarcoding is used in surveillance. Deployment of these two methods is, however, a long way ahead. This is due to the drawbacks associated with these methods, such as nonsensitivity of the metagenomics method compared to quantitative PCR (49, 50). Nonetheless, use of various enrichment methods as well as methods to deplete the host organism would generally improve the sensitivity of this method. Another existent limitation of large-scale metagenomics is the difficulty in associating the detected virus with its host. Metabarcoding, as applied in this study, helps to narrow down the possible hosts of these viruses. However, further improvement of this method is required, as highlighted above, so as to overcome the challenge of associating the individual virus detected to one of the possible insect species. Therefore, future studies will benefit from using viral enrichment methods, as well as methods like the co-occurrence networks, to infer the insect hosts associated with the identified viruses.

## MATERIALS AND METHODS

### Collection and sorting of midge specimens.

The specimens used in this study were collected in 2016 from some of Kenya’s arid and semiarid lands (ASAL), including Baringo, Kacheliba, Turkana, and Isiolo. These are livestock-rearing areas, and the climatic conditions allow for a high density of midges (51). Specimens were collected using CDC light traps (John W. Hock) that were set in the evening (1700 h) and then collected the following morning (0600 h). The traps were placed near resting places for livestock, and they were baited with dry ice held in Igloo containers (52). All the trapped specimens were transported to the site laboratory, where they were sorted and cryopreserved for transportation to the laboratory. The cryopreserved specimens were transported to the laboratory at Kenya Medical Research Institute (KEMRI), where they were identified and pooled into a maximum of 50 specimens per pool based on whether the specimen was a *Culicoides* sp. or unclassified midge, area/site where they were collected, sex, and blood feeding status. The pooled specimens were stored at −80°C until processing.

### Bulk pool preparation.

Individual pools of ≤50 nonfed specimens were first homogenized with Copperhead metal BBs (Crosman, USA) using homogenization medium containing minimum essential medium, with Earle’s salts and reduced NaHCO_3_. The medium was supplemented with 15% heat-inactivated fetal bovine serum (FBS) (Gibco) and 2% each of l-glutamine (Sigma-Aldrich) and antibiotic/antimycotic solution (Sigma-Aldrich). The homogenates were clarified by centrifugation at 10,000 × *g* for 10 min. To prepare samples for metagenomics and metabarcoding analysis, bulk pools were created for each of the 5 regions; Turkana, Baringo, Budalangi, Isiolo, and Kacheliba. The total number of specimens processed for each site included 1,063 from Turkana, 892 from Baringo, and 640, 600, and 156 from Isiolo, Budalangi, and Kacheliba, respectively (Table 2). For metagenomics, all the individually clarified supernatants from the different pools were combined to create five bulk pools for each of the regions. These 5 bulk pools were mixed by vortexing then used for viral RNA extraction. For metabarcoding, the individual pellets from each of the pool of ≤50 specimens were combined for each of the regions to make 5 bulk pools containing the combined crude mixture (Table 2). Homogenization medium, described above, was then added to these bulk pools, and further crushing was performed to ensure adequate mixing of the combined homogenates. The crude mixture was used for DNA extraction.

### Metagenomics. (i) Viral RNA extraction.

The clarified supernatant of the 5 individual bulk pools was passed through 0.22-μm filters to remove excess host “contaminants” and any bacteria while concentrating the viral particles. In preparing the 5 samples, one extraction blank and two positive samples were included as controls. The two positive-control samples were dengue virus type 2 isolates that had been amplified in Vero cells. RNA extraction was performed using a QIAamp viral RNA minikit (Qiagen, Germany) following the manufacturer’s recommended protocol. RNA was quantified using a Nanodrop 2000 spectrophotometer (Thermo Fisher Scientific, USA) and Qubit RNA 2.0 fluorometer using the Qubit RNA HS assay kit (Invitrogen, USA). The RNA was then prepared for Illumina library preparation.

### (ii) Illumina library preparation.

Libraries for sequencing were prepared using TruSeq stranded mRNA kit (Illumina, USA), following the manufacturer’s recommended protocol with modification to exclude the poly(A)-containing mRNA purification steps. Briefly, reverse transcription on ∼25 ng/μl of RNA was achieved by using Superscript III reverse transcriptase (Invitrogen, USA) and random hexanucleotide primers (Invitrogen, USA). This was followed by second-strand synthesis using DNA polymerase I and RNase H, provided with the library preparation kit. Purification was then performed using AMPure XP beads (Beckman Coulter, USA) after which the purified double-strand cDNA fragments were end repaired by adding a single A nucleotide to the 3′ end of the blunt fragments, to prevent the formation of chimeras and improve adapter ligation efficiency. Ligation of the adapters was performed, and the products were purified and enriched by PCR to create the final library. Libraries were normalized and pooled before loading. Sequencing was carried out using the MiSeq reagent kit V3 (Illumina, USA), in a 600-cycle sequencing format.

### (iii) Sequence analysis.

Raw sequence reads were initially subjected to cleaning using Trim Galore v0.6.5 to remove adapters and Prinseq Lite v0.20.4 to remove low-quality reads using the following parameters: minimum length, 50 bp; maximum length, 301 bp; and minimum mean Q score, 30. Further, filtering of the reads was performed by using riboPicker v0.4.3, to remove rRNA sequences by comparing them against the SILVA rRNA database, release 138.1 (53). Paired-end reads were merged using PEAR 0.9.8 (54), and preliminary analysis was performed using the MG-RAST server to classify reads taxonomically. Cleaned reads were assembled *de novo* using the Trinity program (55) with default parameters. The cleaned reads were mapped back to the assembled contigs and filtered to retain only contigs in which at least 90% of bases had 5× coverage (56). Contigs that met this criterion were first compared to the NCBI viral database using the BLASTx program. Potential viral contigs were further compared to the entire NCBI nr database using the BLASTx program to filter out all nonviral sequences. Finally, as a control step to test false positives that might have occurred due to index hopping and carryover contamination, sequence reads belonging to the positive controls and the negative control were mapped against the viral contigs obtained using the Burrows-Wheeler Aligner (BWA-MEM) v 0.7.17. No contaminant contigs were identified during this step. Sequences that were confirmed to be of viral origin were translated, and ORF predictions were performed on them using the Expasy server (57). Phylogenetic reconstructions were performed based on the RNA-dependent RNA polymerase (RdRp) gene. To ensure meaningful depiction of the evolutionary relationships of the newly discovered viruses, their closest RdRp homologs were downloaded from GenBank and used as reference sequences in the phylogenetic analysis. Maximum-likelihood phylogenies were inferred using iqtree (58), with simultaneous evaluation of the best model and tree searching being performed based on 1,000 bootstrap estimates and 1,000 approximate-likelihood-ratio tests. The inferred phylogenies were visualized in Figtree v1.4.4.

### Metabarcoding. (i) DNA extraction.

DNA extraction was performed on the bulk pools of the crude homogenates using a QIAamp DNA extraction kit (Qiagen, Germany), according to the manufacturer’s instructions. Two extraction blanks were included during the extraction process, and subsequently used during PCR and sequencing. The extracted DNA was quantified using Nanodrop 2000 (Thermo Fisher Scientific, USA). Amplification of the COI gene was then carried out on <1 μg of extracted DNA, using the universal pair of primers for metazoan invertebrates LCO1490/HCO2198 (59). These primers amplify an approximately 710-bp region of the COI gene of arthropod vectors. COI amplicons were generated from a 25-μl PCR containing 12.5 μl AmpliTaq Gold 360 master mix (Applied Biosystems, USA), 9.5 μl DNase/RNase-free water, and 0.5 μl each of the forward and reverse primers at 25 μM. The PCR cycling conditions were set as follows; initial denaturation at 95°C for 10 min, 35 cycles of 95°C for 30 s, 49°C for 30 s, 72°C for 30 s, and a final extension of 72°C for 7 min.

### (ii) MinION library preparation.

The COI amplicons were first purified using AMPure XP beads (Beckman Coulter, USA). The purified products were quantified using a Qubit dsDNA HS assay kit (Invitrogen, USA) with a Qubit fluorometer 2.0. Based on the concentration of the quantified products, the volume of PCR products that yielded 200 fmol was determined and used as starting material for MinION library preparation. Library preparation was carried out using a ligation sequencing kit (SQK-LSK109), following the manufacturer’s protocol with the exclusion of the DNA fragmentation step. Briefly, 200 fmol of the purified products were end repaired using a NEBNext Ultra II end repair and dA-tailing module (New England Biolabs [NEB], UK). The end-repaired DNA for each sample was individually barcoded using Native Barcoding Expansion 1-12 (EXP-NBD104), which was achieved with the use of NEB Blunt/TA ligase master mix (NEB, UK). An equal amount from each of the 200-fmol barcoded libraries was combined into a single pool, which was then purified with AMPure XP beads (Beckman Coulter, USA). Adapter ligation of the purified library was done with NEBNext quick ligation module (NEB, UK) and the libraries were further purified using AMPure XP beads, with a final wash of the beads being carried out using short fragment buffer (SFB) provided with the SQK-LSK109 kit. The final library was loaded onto the flow cell (FLO-MIN106D) and sequenced using the workflows provided in the MinKNOW software.

### (iii) Sequence analysis.

Base-calling and demultiplexing were performed on the MinION Mk1C device using Guppy. Sequencing reads were quality filtered with Nanofilt v2.8.0 (60), in order to retain only the higher-quality reads with a read quality score of ≥10 as recommended by Nanopore (https://github.com/nanoporetech/ont_tutorial_basicqc). Reads that were longer or shorter than the expected length of approximately 710 bp (with a 150-bp buffer) were also filtered. In addition, sequences that were identical to those detected in the extraction blanks were removed. Error correction of the sequence reads was performed using isONclust v0.0.6 and isONcorrect v0.0.8 (61, 62), using default parameters with the –*ont* flag. The corrected reads were resampled to approximately 11,000 reads per sample, using rasusa v0.5.0 (63). Read clustering, consensus sequence generation, and determination of the number of reads supporting each consensus sequence were carried out using IsoCon v0.2.5.1 (64). IsoCon treats reverse complements and sequence duplicates of various lengths as different. Therefore, these were further removed by performing clustering of the consensus sequences using cd-hit-est (65) with a 98.9% similarity threshold, which is the lowest accuracy of error-corrected nanopore reads (62). Taxonomic assignation of the consensus sequences was performed in the MIDORI server using RDPClassifier with COI reference sequence database (66, 67). This was further validated by searching against the NCBI nr database, in order to determine the lowest classification of each of the consensus sequence. We excluded any OTUs that were not classified as belonging to an expected invertebrate phylum. To increase the reliability of the identified OTUs, singletons were removed and only OTUs that were supported by ≥10 sequences were retained. Further, invertebrate species supported by less than 1% of the total sequences in each site were also removed from the final analysis.

### Ethical approval.

Ethical approval to carry out this study was obtained from the Kenya Medical Research Institute’s Scientific Ethics Review Unit (SERU), under protocol number KEMRI SSC 3693.

### Data availability.

The sequences of the viruses identified in the study have been submitted to GenBank under accession numbers MZ078285 to MZ078300. The consensus sequences of the cytochrome oxidase subunit 1 gene (COI) for the specimens in the study are also available in GenBank under the accession numbers MZ227639 to MZ227812 and OK357592 to OK357601.
